# Discovery and Validation of Methylation Biomarkers for Ulcerative Colitis Associated Neoplasia

**DOI:** 10.1093/ibd/izy119

**Published:** 2018-05-14

**Authors:** Andrew D Beggs, Jonathan James, Germaine Caldwell, Toby Prout, Mark P Dilworth, Phillipe Taniere, Tariq Iqbal, Dion G Morton, Glenn Matthews

**Affiliations:** Institute of Cancer and Genomic Science, University of Birmingham

**Keywords:** ulcerative colitis associated dysplasia, methylation, biomarkers

## Abstract

**Background and aims:**

Ulcerative colitis (UC) is associated with a higher background risk of dysplasia and/or neoplasia due to chronic inflammation. There exist few biomarkers for identification of patients with dysplasia, and targeted biopsies in this group of patients are inaccurate in reliably identifying dysplasia. We aimed to examine the epigenome of UC dysplasia and to identify and validate potential biomarkers

**Methods:**

Colonic samples from patients with UC-associated dysplasia or neoplasia underwent epigenome-wide analysis on the Illumina 450K methylation array. Markers were validated by bisulphite pyrosequencing on a secondary validation cohort and accuracy calculated using logistic regression and receiver-operator curves.

**Results:**

Twelve samples from 4 patients underwent methylation array analysis and 6 markers (GNG7, VAV3, KIF5C, PIK3R5, TUBB6, and ZNF583) were taken forward for secondary validation on a cohort of 71 colonic biopsy samples consisting of normal uninflamed mucosa from control patients, acute and chronic colitis, “field” mucosa in patients with dysplasia/neoplasia, dysplasia, and neoplasia. Methylation in the beta-tubulin *TUBB6* correlated with the presence of dysplasia (*P* < 0.0001) and accurately discriminated between dysplasia and nondysplastic tissue, even in the apparently normal field mucosa downstream from dysplastic lesions (AUC 0.84, 95% CI 0.81–0.87).

**Conclusions:**

Methylation in TUBB6 is a potential biomarker for UC- associated dysplasia. Further validation is needed and is ongoing as part of the ENDCAP-C study.

## INTRODUCTION

Ulcerative colitis (UC) is a systemic autoimmune inflammatory disorder, primarily targeted at the colon and rectum, leading to widespread transmural mucosal inflammation. It is associated with an increased risk of cancer^1^ and a recent meta-analysis demonstrated that in population-based cohorts, cancer risk increases 2.4-fold, with male sex, young age at diagnosis, and extensive colitis further increasing the risk.^2^

UC-associated cancers arise in a similar fashion to sporadic carcinomas, however, they arise through an inflammation-dysplasia-carcinoma sequence^3, 4^ that leads to accelerated carcinogenesis. The risk of cancer increases exponentially with duration of disease and has led to screening guidelines that take account of duration of symptoms.

The British Society of Gastroenterology guidelines^5^ recommend that a single surveillance colonoscopy should be performed within 8 years of onset of symptoms, followed by 5 yearly in the second decade, 3 yearly in the third decade, and yearly in the fourth decade. It also is recommended that as part of these colonoscopies approximately 2–4 serial biopsies be taken every 5-10 cm within the colon. This represents a considerable burden for the screening endoscopist, although technologies such as chromoendoscopy^6^ and narrow band imaging^7^ have been used, to varying degrees of success. The use of random biopsies, even where they are taken frequently, may also reduce the chance of successful detection of UC-associated dysplasia or cancer.^8^

A need exists for biomarkers that can reliably detect UC-associated dysplasia and cancer within the colon as part of a screening program.^9^ A number of different approaches and technologies have been used to find an effective biomarker. Bronner et al^10^ utilised fluorescent in situ hybridisation (FISH) of fresh frozen colonic biopsy specimens, finding 100% sensitivity and 92% specificity in discriminating patients who went on to develop neoplasia from those who did not.

Leedham et al^11^ investigated mutational spectrum in UC-associated dysplasia and found that TP53 mutation was most frequent, with KRAS driver mutations being found in a subset of tumours. They also demonstrated that the lesions seen in UC-associated neoplasia were monoclonal in origin, with a driver mutation leading to multiple clones possessing the same mutation. Risques et al found that shortening of telomeres, associated with cellular senescence^12^ in a field effect, seemed to indicate which areas of UC-associated dysplasia were likely to progress to cancer.

Other genetic markers for high risk UC-associated dysplasia that have been studied include chromosomal instability and microsatellite instability. Rubin et al^13^ studied the rates of aneuploidy in patients with UC-associated dysplasia, finding an association between aneuploidy in biopsy specimens and progression to neoplasia, however this association has not been consistently reported by other studies.^14^ Microsatellite instability (MSI) also has been studied as a potential marker, however no strong correlations between MSI and progression to neoplasia has been found.^15, 16^

It is obvious that current markers for high risk UC-associated neoplasia are lacking in accuracy. Analysis of methylation, an epigenetic modification to DNA, has several advantages. Firstly, DNA methylation is a stable change that is detectable in formalin fixed, paraffin-embedded biopsy samples.^17^ Secondly, disease associated regions of methylation change tend to occur in longer stretches of DNA than point mutations^18^ and in fields of change within affected tissues, allowing easier assay design and less need for targeted biopsies.

We therefore aimed to study the changes seen in DNA methylation in high risk UC-associated dysplasia to develop potential biomarkers for this disease.

## METHODS

### Patients and Ethical Approval

Patients were recruited from the University Hospital Birmingham inflammatory bowel disease (IBD) clinic using an in-house IBD database to identify patients. For patients with neoplasia, patients were included in the study if they had UC of greater than 8 years in duration, had developed UC-associated neoplasia, had a proctocolectomy for UC-associated neoplasia with frozen material taken, and had archival histological material (tumor and paired normal) available for analysis. Patients were excluded if they did not have histological material available for analysis or the duration of their disease was less than 8 years. Acute colitis was defined as a first presentation of colitis, and chronic colitis was defined as colitis of greater than 8 years in duration. All patients with both “acute” and “chronic” colitis were sampled when they were receiving (at the most) only 5-ASA therapy for their colitis, rather than immunosuppressive agents that may bias methylation measurements.

Patients with chronic inflammation caused by UC without the development of neoplasia also were recruited to serve as nonneoplastic but inflamed controls. This was to exclude biomarkers that may have been associated with inflammation rather than driving tumoriogenesis.

The study was carried out with full ethical approval from the South Birmingham Research Ethics Committee (08/H1207/104).

### Sample Collection, Extraction, and Quantification

Tissue blocks were retrieved from histology archives, and associated H&E sections were reviewed by a consultant histopathologist (Phillipe Taniere) to ensure accuracy of histological diagnosis. For the discovery set, frozen tissue was obtained at the time of proctocolectomy by opening the specimen and representative samples of tumor and normal mucosa were obtained. Normal mucosa was obtained at the maximal possible distance from the tumor specimen. Histological type was confirmed by frozen sectioning, and all tumor material was confirmed to be adenocarcinoma. Dysplasia was categorized according to the methodology of Riddell et al^19^ as either “high grade” or “low grade”. This was snap frozen in liquid nitrogen then stored at -80 C until needed. For archival specimens, blocks were sectioned into 10μM sections and placed on slides. Needle macrodissection using white light microscopy comparing to a representative H&E section was used to enhance for tumor content. For DNA extraction of both sets, samples were immersed in 300 uL of buffer ATL (Qiagen Ltd, Manchester, UK) and 20μL of 20mg/μL Proteinase K (VWR Jencons, Lutterworh,UK). Samples were incubated overnight in a tissue oven, spun down to form a wax plug that was then punctured (for FFPE samples), and the lysate retrieved. This was cleaned and purified using a Qiagen DNeasy Blood & Tissue kit (Qiagen Ltd, Manchester UK). Extracted DNA was quantified for purity using a Nanodrop ND-2000 spectrophotometer and for quantity using a Qubit fluorimeter. If samples did not pass a quality threshold of A260/280 >1.8 they were reextracted. DNA was stored at -80 C until ready for use.

### Methylation Microarray Discovery

To quantify methylation across the whole genome, the Illumina HumanMethylation450 array system was used on fresh tissue from the first part of the study. This is an oligonucleotide-based microarray platform that has over 458,000 probes targeted at CpG dinucleotides selected by an international consortium of epigenetics researchers to cover gene promoter regions, differentially methylated regions (DMRs), and other regions of interest.

One microgram of extracted DNA was bisulphite converted using the Zymo EZ-DNA Methylation kit with a modified protocol suitable for use on Illumina microarrays. A standard amplification, hybridization, labeling, and wash procedure was carried out by the Core Genomics Facility at the Wellcome Trust Centre for Human Genetics, University of Oxford. Microarrays were scanned on an Illumina iScan array scanner and detected intensities were converted to IDAT files and exported for further use.

Exported intensity data were analyzed using a combination of limma/Bioconductor and the ChAMP pipeline for methylation array analysis.^20^ Data were imported into R 2.15.1 and were filtered to remove all probes that had failed the detection threshold (*P* > 0.05). Quality control plots were also produced and any samples failing lllumina standard QC were excluded. Probes were then normalized to adjust for Type 2 bias using BMIQ normalization, underwent SVD identification for components of variation, and batch correction using COMBAT. Top differentially methylated probes were called using a 3 level regression and eBayes shrinkage of moderated t-statistics using limma and DMRs called using DMRHunter. Copy number variation was called using the copy number function of the ChAMP package.

### Validation

To provide validation samples, the IBD database was interrogated to provide a further cohort of samples for validation using bisulphite pyrosequencing. Targets for validation were identified from the top hits from the CHaMP analysis. The Illumina probe identifier for each hit was retrieved and genomic coordinates for the relevant CpG dinucleotide were identified from the HumanMethylation450 manifest file.

A primer set for bisulphite pyrosequencing was designed flanking the CpG dinucleotide of interest using Qiagen PyroMark 2.0 software using standard conditions. If a primer set could not be designed using standard conditions, the relevant conditions (Tm, amplicon length) were adjusted until a set could be designed. A maximum amplicon length of 250 bp was set as the validation samples originated from FFPE samples and our previous experiences in FFPE primer design had demonstrated that this was the maximum achievable primer sequence for this type of sample. Designed primers were biotinylated in either the forward or reverse direction, depending on design characteristics and primers were obtained from Sigma-Aldrich (Primer sequences available on request).

Obtained forward and reverse primers were diluted to 20 uM and used in a gradient PCR reaction using the Qiagen Pyromark PCR kit under standard conditions in a reaction volume of 25 uL to obtain optimum Tm. For each pyrosequencing reaction, 20 ng of bisulphite treated DNA in a volume of 2uL was used in a 25 uL reaction using the Qiagen Pyromark PCR kit under standard conditions with the observed Tm. Products were then cleaned and pyrosequenced on a Pyromark 96 ID machine using a 1:100 dilution of 20 uM sequencing primer. Percentage methylation values were calculated using standard default software settings on the Pyromark Q-CpG software package. All reaction plates were run with 100% methylated (generated with MSSl treatment of genomic DNA) and unmethylated (generated by whole genome amplification using Qiagen Repli-G kit) DNA.

### Statistical Analysis

To predict accuracy and other metrics in the identified markers, methylation data were exported to Stata 12.1 (StataCorp, Texas, USA) as percentages. As methylation data were likely to be nonnormally distributed, a Wilcoxon rank sum test was performed on case-control data. To compare differences between tissue types, analysis of variance (ANOVA) testing was carried out. To estimate test accuracy a logistic regression model using outcome (cancer/no cancer) as the dependent variable and using percentage methylation for each marker was used as the independent variables. Markers identified as significant (*P* < 0.05) were then subjected to a sensitivity analysis using ROC curves to identify a cutoff of methylation to identify neoplasia to optimize sensitivity and specificity. This threshold was then modeled using the *diagt* function of Stata, correcting for a population prevalence of UC-associated dysplasia of ~3%.

## RESULTS

### Patients

For the discovery cohort, a cohort of 4 patients were obtained with tumor (adenocarcinoma) material and matched normal mucosa (Table 1), giving a total of 8 samples. A further 4 samples of normal mucosa in patients with chronic UC were obtained, giving 12 samples that were run successfully on the HumanMethylation450 array. For the patients with UC-associated neoplasia 75% (3/4), patients were male with an average age of 48 years. For the chronically inflamed normal mucosa, 50% (2/4) patients were male with an average age of 41 years. The duration of UC in these patients is shown in Table 1.

**Table 1:** Table of Recruited Patients to Study (MICROARRAY)

Code	Sample	Tissue Type	Location	Sex	Age	Group
1	DA1C	Tumor	Ascending colon	M	38	
2	DA3B	Matched	Hepatic Flexure	M	38	
3	DK1A	Tumor	Sigmoid	M	33	
4	DK3A	Matched	Transverse	M	33	
5	ES3B	Tumor	Descending	M	72	
6	ES2B	Matched	Transverse	M	72	
7	NBT1	Tumor	Rectum	F	50	
8	NB2NA	Matched	Transverse	F	50	
9	8IBD33	Normal	Descending	M	38 or 28	Control (<1 yr)
10	9695	Normal	Rectum	M	51	Control (3 yr)
11	9693	Normal	Rectum	F	32	Control (6 yrs)
12	9730	Normal	Rectum	F	44	Control (<1 yr)

For the validation cohort, a total of 71 samples from 71 patients were used. A decision was made to obtain a single sample from each patient to attempt to reduce bias. These samples were biopsy samples consisting of acute colitis (n = 16), UC-associated cancer (n = 11), chronic (>8 years) colitis (n = 9), UC-associated dysplasia (n = 9, all high grade dysplasia), normal mucosa downstream of neoplasia (n = 19), and completely normal mucosa from unaffected controls (n = 7). The gender and age data of these samples are shown in Table 2. To expand and enhance the dataset, we used Human Methylation450 array data from the TCGA (The Cancer Genome Atlas) project, with 245 cases of colorectal cancer and 38 normal mucosa controls.

**Table 2:** Table of Validation Cohort

Summary	Median Age	Range Age	Number of Samples	Male:Female	Pan:Left:Proc (U)	Dur Med
Control	44	24–68	6	5:1	NA	NA
Low Risk (Acute)	40	20–66	17	6:11	2:6:9	3
Intermediate (Chronic)	45	21–73	23	10:13	14:3:6	17
Field mucosa	57	38–78	19	15:4	11:2:1 (3)	11
UC-Dys	63.5	43–73	10	6:4	5:1:2 (2)	10
UC-Can	54	26–67	16	14:2	12:0:2 (2)	10

### Differentially Methylated Positions (DMP)

A multilevel analysis of change in methylation was carried out, assuming that methylation would change between chronically inflamed mucosa, tumor-associated mucosa, and tumor itself as part of a field effect. This analysis revealed 12,412 diffferentially methylated probes with a Manzel- Haenszel adjusted *P*value of < 0.05. The top 20 probes are shown in Table 3.

**Table 3:** Probe of Top Differentially Methylated CpGs Within Cohort Including Functions, Significance and Direction of Change (ID = Illumina Probe ID, Chr = Chromosome, logFC = log fold change, T = t-statistic, *P*value = unadjusted *P*-value, adj.p.Val = adjusted *P*value, B = Bayes Factor)

Rank	ID	Chr	Gene	logFC	t	*P*Value	adj.*P*Val	B
1	cg08626004	19	*GNG7*	-4.32	-12.2	7.92E-09	0.0011	8.97
2	cg03507241	18	*TUBB6*	-0.31	-13.5	2.62E-08	0.0013	8.86
3	cg25848557	1	*VAV3*	4.97	19.0	2.64E-09	0.0014	8.30
4	cg03280624	19	*ZNF583*	5.82	18.0	4.39E-09	0.0016	8.11
5	cg12035092	2	*KIF5C*	3.99	16.3	1.18E-08	0.0016	7.69
6	cg12863545	17	*PIK3R5*	4.15	15.5	1.89E-08	0.0017	7.47
7	cg00796360	19	*ZNF583*	4.05	15.1	2.46E-08	0.0017	7.35
8	cg24347663	2	*ADD2*	4.23	14.8	3.06E-08	0.0018	7.24
9	cg22260952	12	*CHST11*	3.38	13.9	5.63E-08	0.0024	6.93
10	cg23977631	2	*LONRF2*	4.75	13.4	7.67E-08	0.0025	6.76
11	cg13644629	19	*ZNF549*	4.22	13.3	8.30E-08	0.0025	6.72
12	cg26998044	17	*PIK3R5*	3.86	13.1	9.82E-08	0.0027	6.63
13	cg13916740	19	*ZNF582*	4.55	13.1	1.02E-07	0.0027	6.60
14	cg19538890	4	*RNF150*	2.91	9.3	2.38E-07	0.0312	6.58
15	cg27230784	17	*LOC728392*	3.82	13.0	1.08E-07	0.0027	6.57
16	cg27009208	19	*ZNF583*	4.72	12.9	1.12E-07	0.0027	6.55
17	cg23967742	10	*CH25H*	4.32	9.0	3.68E-07	0.0312	6.24
18	cg12936797	4	*RNF150*	2.67	8.8	4.33E-07	0.0312	6.12
19	cg08071282	16	*TPSD1*	-1.96	-8.5	6.70E-07	0.0312	5.78
20	cg06168875	19	*NANOS3*	-2.25	-8.5	6.84E-07	0.0312	5.76

The top ranked CpG, cg08626004 (logFC = -4.32, B = 8.97) lies within a CG-rich region of exon 5 of GNG7 (G-protein subunit gamma 7), a membrane bound GTPase linked to 7-TM receptors. The next ranked CpG, cg03507241 (logFC = -0.31, B = 8.86) lies within the promoter region of TUBB6 (Tubulin Beta 6 class V) that codes for a gene that acts as 1 of the tubulin scaffold components of microtubules. The third ranked CpG, cg025848557 lies within intron 1 of VAV3-AS1 (VAV3-antisense 1), a noncoding RNA. The fourth ranked CpG, cg03280624 lies within the promoter region of ZNF583, a zinc finger-related transcription factor. The fifth ranked CpG, cg12035092 lies within exon 1 of KIF5C, a kinesis heavy chain subunit. The sixth ranked CpG, cg12863545 lies within the promoter of PIK3R5, a PI3 kinase-related gene.

### Differentially Methylated Regions

A similar analysis was carried out for diffentially methylated regions using the dmr.lasso function of ChAMP (Table 4) examing the differences between chronically inflamed mucosa, tumor-associated mucosa, and tumor itself. The top differentially methylated region was within SGCE (Sarcoglycan epsilon), a transmembrane protein that is a component of the dystrophin-glycoprotein complex. The second highest differentially methylated region was within SOX2OT (SOX2 overlapping transcript), a long noncoding RNA that overlaps the coding region of the SOX2 gene and has been shown to regulate SOX2 expression.^21, 22^

**Table 4:** Table of Differentially Methylated Regions (DMR) from Methylation Array Data (Chr = Chromosone, DMR start – start position of DMR in base pairs, DMR End – end position of DMR in base pairs, DMR size = size of DMR in base pairs, DMR P = significance of DMR)

Rank	Chr	Gene	DMR start	DMR End	DMR size	DMR P
1	7	*SGCE*	94284472	94285349	878	2.96E-08
2	3	*SOX2OT*	181428147	181428730	584	5.74E-07
3	3	*DGKG*	186079969	186080309	341	6.14E-06
4	17	*HOXB3*	46650083	46653384	3302	6.79E-06
5	1	*RXRG*	165414114	165414644	531	1.67E-05
6	4	*RNF150*	142054752	142054884	133	1.79E-05
7	17	*LOC404266*	46681474	46683967	2494	2.08E-05
8	11	*PTPRCAP*	67204926	67206552	1627	0.000102554
9	6	*LY6G6D*	31684923	31686011	1089	0.000105019
10	17	*LOC100133991*	43339397	43339594	198	0.00011108

### Copy Number Variation

Copy number was called for all 12 samples successfully using the ChAMP package. In the tumor group, a recurrent deletion of variable length was seen at the very end of the q-arm of chromosome 5 in 3 out of 4 tumors (Chr 5:178017667-180876320). This CNA is frequently seen in colorectal cancer, and has been reported at a high frequency in UC-related cancers.^23^ A recurrent variable length gain of the p-arm of chromosome 5 was also seen, which has also been previously reported in UC- associated cancer^23^

In agreement with previous studies, a diverse pattern of copy number alteration was seen in both normal mucosa downstream of a tumor and in matched chronically inflamed mucosa. In the matched normal mucosa from downstream of the tumor, a recurrent copy number loss was seen in 3 out of 4 samples in chromosome 17q, localized to a 2 mb region (Chr17:40169693-41993127). In the chronically inflamed normal mucosa, recurrent chromosomal loss was seen in chromosomes 1,2,6,10,11,17, 19, and 20 where recurrent loss was defined as >75% of samples.

### Validation of Observed Methylation Changes

To validate changes seen in methylation, bisulphite pyrosequencing was carried out on the validation set of samples as described in methods. Based on the observed DMPs, the following assays were designed: *ZNF583*, *GNG7*, *PIK3R5*, *TUBB6,* and *KIF5C*. An attempt was made to design a primer set to amplify VAV3, but this region was found to be very GC rich (GC content >85%) making amplification very challenging and, therefore, further study of this region was stopped. To simplify analysis, sample groups were consolidated into 2 groups for the purposes of the initial analysis, control (which included normal colon, acute colitis, and chronic colitis) and case (which included field mucosa, dysplasia, and cancer). Validation on the remaining panel demonstrated nonsignificance on all markers in cases versus controls except TUBB6 (Table 5), which demonstrated a median methylation of 11% in controls (IQR 4) and 37% (IQR 42) in cases.

**Table 5:** Table of Median Methylation in Genes to be Validated

Gene	Median Control (IQR)	Median Methylation Dysplasia (IQR)	*P*value (Wilcoxon)
GNG7	75 (15.5)	77 (15.5)	0.86
KIF5C	2.5 (6)	0 (6)	0.05
PIK3R5	2 (8.5)	0 (10)	0.32
TUBB6	11 (4)	37 (42)	<0.0001
ZNF583	2 (0.5)	5.5 (5.5)	0.06

**FIGURE 1.**
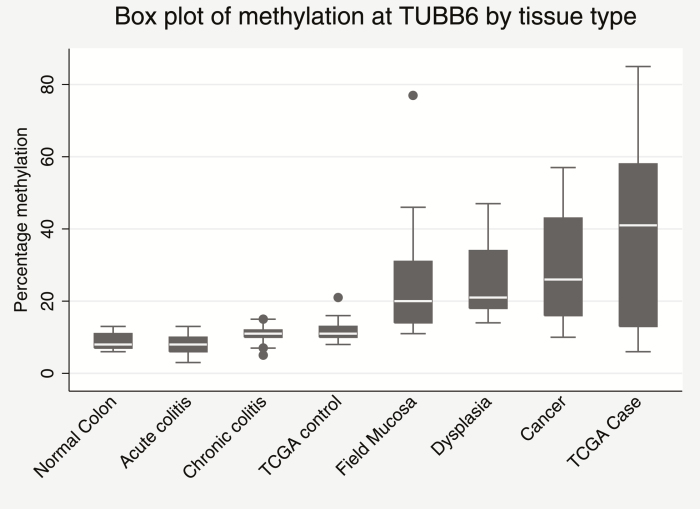
Box plot of percentage methylation as determined by bisulphite pyrosequencing at TUBB6 by tissue type.

Further study of TUBB6 by tissue group demonstrated a significant (ANOVA *P* = 0.0043, F = 3.81) progression in methylation from normal mucosa, where methylation was at its lowest, through acute inflammation to dysplasia and neoplasia where methylation was as its highest (Fig. 1). Interestingly, methylation within the “field mucosa”, ie, the mucosa downstream of a dysplastic or neoplastic lesion also had increased methylation as compared to control suggesting a field effect in these patients. Due to limited sample material, we were unable to perform immunochemistry for *TUBB6* expression to compare to methylation levels, however an analysis of the TCGA dataset for colorectal cancer suggested no correlation between methylation and expression of TUBB6 (Supplementary figures 1).

Modeling of the association between TUBB6 methylation levels and presence of invasive disease (taken as either “field” mucosa, dysplasia, or cancer) using logistic regression found that a threshold of 17% methylation was sufficient to discriminate invasive disease correcting for a population prevelance of UC-associated dysplasia of 3% (AUC 0.84, 95% CI 0.81–0.87), Supplementary figure 2. This gave a final post hoc test sensitivity of 70.1%, specificity of 98.6%, positive predictive value of 60.3%, negative predictive value of 99.1% and a Youden index of 0.68.

## CONCLUSIONS

In this study, we have carried out an exploratory analysis of the changes observed in methylation in tissue samples from patients with UC in the progression from normal mucosa, to disease associated “normal” mucosa, to dysplasia/neoplasia. We have then subsequently successfully validated differential methylation of *TUBB6* as being a biomarker for progression in UC to invasive disease. To our knowledge, this is the first study that has utilized the power of epigenome-wide analysis to identify potential biomarkers of progression in UC . Abnormal methylation in cancer (both hyper- and hypomethylation) has been demonstrated to be adversely associated with survival across multiple cancer types.^24–26^

Our study has a significant weakness, such that the initial cohort consists of 4 patients with paired tumor and associated normal “field” mucosa with normal mucosa obtained from normal control mucosa. This could potentially lead to test inflation caused by small sample size leading to a high false discovery rate, and this probably accounts for our observation that 4 out the 5 markers did not pass statistical validation. However, 2 of them - KIF5C and ZNF583 – only just did not reach significance and were therefore potential markers but were dropped because their observed methylation values included 0 values that would make test failures difficult to discriminate from true results.

However, our top ranked marker, TUBB6 validated in our independent cohort to a high significance level and demonstrated its possibility as a potential biomarker for UC-associated dysplasia. This demonstrates that it is possible to demonstrate statistically significant biomarkers from small genome-wide datasets given careful statistical analysis, making this a cost effective way to perform these studies in other diseases.

The function of our identified markers remains obscure and ideally a biomarker should have a functional relevance to the disease being studied. Two genes that almost reached significance―KIF5C and ZNF583―have functions not directly related to UC pathogenesis or progression. KIF5C (kinesin family member 5C) is a kinesin heavy chain subunit involved with protein trafficing within the central nervous system and has been linked to intellectual disability.^27^ Little is known of the function of ZNF583 (Zinc Finger 583) beyond its description as a zinc finger protein,^28^ making it presumably involved in transcriptional regulation.

TUBB6 codes for Tubulin beta 6, a tubulin scaffold protein associated with the formation of microtubules that are part of the cytoskeleton.^29^ A recent celluar genome-wide associated study highlighted the importance of differential expression^30^ of TUBB6 in the promotion of inflammatory cell death, known as pyroptosis. The study demonstrated that increased expression of TUBB6, in this case caused by an intragenic single nucleotide polymorphism, lead to decreased pyroptosis. This has potential associations with the mechanisms of cell death in UC , as deficiencies in commensal-induced pyroptosis has been shown to increase the severity of UC in a murine model.^31^ Variable TUBB6 expression has also been seen in other malignancies such as nonsmall cell lung cancer^32^ and prostate, ovary, and breast cancer.^33^ Some evidence has been demonstrated in cell lines by Mariani et al^34^ that TUBB6 expression is partially controlled by androgen receptor status, with women showing higher expression of TUBB6 than men. However, our study is concerned with methylation of *TUBB6* and the relationship between expression and methylation is complex and therefore it is difficult to understand whether there is an impact of gender on methylation of *TUBB6* as we have not shown any particular bias. Potentially deficient microtubules could be stabilized by an agent such as paclitaxel^35^ offering a potential target for “high risk” colitis.

In conclusion, we have identified and validated a potential biomarker of UC-associated dysplasia in the form of abnormal methylation of TUBB6, identified by whole epigenome analysis. Further validation is essential and this marker will form part of a marker panel in the prospective clinical trial module of epigenetic biomarkers in the NIHR funded ENDCAP-C study.

## SUPPLEMENTARY DATA

Supplementary data is available at *Inflammatory Bowel Diseases* online.

Supplementary Material

## Notes

Supported by: Andrew D. Beggs received salary support from the Wellcome Trust (102732/Z/13/Z) and Cancer Research UK (C31641/A23923) during this grant.

## References

[1] Eaden JA, Abrams KR, Mayberry JF The risk of colorectal cancer in ulcerative colitis: a meta-analysis. Gut. 2001;48:526–35. 11247898 10.1136/gut.48.4.526 PMC1728259

[2] Jess T, Rungoe C, Peyrin-Biroulet L Risk of colorectal cancer in patients with ulcerative colitis: a meta-analysis of population-based cohort studies. Clin Gastroenterol Hepatol. 2012;10:639–45. 22289873 10.1016/j.cgh.2012.01.010

[3] Kulaylat MN, Dayton MT Ulcerative colitis and cancer. J Surg Oncol. 2010;101:706–12. 20512947 10.1002/jso.21505

[4] Kinugasa T, Akagi Y Status of colitis-associated cancer in ulcerative colitis. World J Gastrointest Oncol. 2016;8:351–7. 27096030 10.4251/wjgo.v8.i4.351 PMC4824713

[5] Eaden JA, Mayberry JF; British Society for Gastroenterology; Association of Coloproctology for Great Britain and Ireland Guidelines for screening and surveillance of asymptomatic colorectal cancer in patients with inflammatory bowel disease. Gut. 2002;51(Suppl 5):V10–2. 12221032 10.1136/gut.51.suppl_5.v10 PMC1867735

[6] Rutter MD, Saunders BP, Schofield G, et al Pancolonic indigo carmine dye spraying for the detection of dysplasia in ulcerative colitis. Gut. 2004;53:256–60. 14724160 10.1136/gut.2003.016386 PMC1774934

[7] Ignjatovic A, East JE, Subramanian V, et al Narrow band imaging for detection of dysplasia in colitis: a randomized controlled trial. Am J Gastroenterol. 2012;107:885–90. 22613903 10.1038/ajg.2012.67

[8] Bopanna S, Roy M, Das P, et al Role of random biopsies in surveillance of dysplasia in ulcerative colitis patients with high risk of colorectal cancer. Intest Res. 2016;14:264–9. 27433149 10.5217/ir.2016.14.3.264 PMC4945531

[9] Thorsteinsdottir S, Gudjonsson T, Nielsen OH, et al Pathogenesis and biomarkers of carcinogenesis in ulcerative colitis. Nat Rev Gastroenterol Hepatol. 2011;8:395–404. 21647200 10.1038/nrgastro.2011.96

[10] Bronner MP, O’Sullivan JN, Rabinovitch PS, et al Genomic biomarkers to improve ulcerative colitis neoplasia surveillance. Am J Pathol. 2008;173:1853–60. 18988798 10.2353/ajpath.2008.080250 PMC2626395

[11] Leedham SJ, Graham TA, Oukrif D, et al Clonality, founder mutations, and field cancerization in human ulcerative colitis-associated neoplasia. Gastroenterology. 2009;136:542–50.e6. 19103203 10.1053/j.gastro.2008.10.086

[12] Risques RA, Lai LA, Himmetoglu C, et al Ulcerative colitis-associated colorectal cancer arises in a field of short telomeres, senescence, and inflammation. Cancer Res. 2011;71:1669–79. 21363920 10.1158/0008-5472.CAN-10-1966 PMC3077943

[13] Rubin CE, Haggitt RC, Burmer GC, et al DNA aneuploidy in colonic biopsies predicts future development of dysplasia in ulcerative colitis. Gastroenterology. 1992;103:1611–20. 1426881 10.1016/0016-5085(92)91185-7

[14] Löfberg R, Broström O, Karlén P, et al DNA aneuploidy in ulcerative colitis: reproducibility, topographic distribution, and relation to dysplasia. Gastroenterology. 1992;102:1149–54. 1551524

[15] Fujiwara I, Yashiro M, Kubo N, et al Ulcerative colitis-associated colorectal cancer is frequently associated with the microsatellite instability pathway. Dis Colon Rectum. 2008;51:1387–94. 18546042 10.1007/s10350-008-9212-9

[16] Løvig T, Andersen SN, Clausen OP, et al Microsatellite instability in long-standing ulcerative colitis. Scand J Gastroenterol. 2007;42:586–91. 17454879 10.1080/00365520601013747

[17] Balic M, Pichler M, Strutz J, et al High quality assessment of DNA methylation in archival tissues from colorectal cancer patients using quantitative high-resolution melting analysis. J Mol Diagn. 2009;11:102–8. 19179456 10.2353/jmoldx.2009.080109 PMC2665859

[18] Witte T, Plass C, Gerhauser C Pan-cancer patterns of DNA methylation. Genome Med. 2014;6:66. 25473433 10.1186/s13073-014-0066-6 PMC4254427

[19] Riddell RH, Goldman H, Ransohoff DF, et al Dysplasia in inflammatory bowel disease: standardized classification with provisional clinical applications. Hum Pathol. 1983;14:931–68. 6629368 10.1016/s0046-8177(83)80175-0

[20] Morris TJ, Butcher LM, Feber A, et al ChAMP: 450k chip analysis methylation pipeline. Bioinformatics. 2014;30:428–30. 24336642 10.1093/bioinformatics/btt684 PMC3904520

[21] Shahryari A, Jazi MS, Samaei NM, et al Long non-coding RNA SOX2OT: expression signature, splicing patterns, and emerging roles in pluripotency and tumorigenesis. Front Genet. 2015;6:196. 26136768 10.3389/fgene.2015.00196 PMC4469893

[22] Askarian-Amiri ME, Seyfoddin V, Smart CE, et al Emerging role of long non-coding RNA SOX2OT in SOX2 regulation in breast cancer. Plos One. 2014;9:e102140. 25006803 10.1371/journal.pone.0102140 PMC4090206

[23] Aust DE, Willenbucher RF, Terdiman JP, et al Chromosomal alterations in ulcerative colitis-related and sporadic colorectal cancers by comparative genomic hybridization. Hum Pathol. 2000;31:109–14. 10665921 10.1016/s0046-8177(00)80206-3

[24] Thompson MJ, Rubbi L, Dawson DW, et al Pancreatic cancer patient survival correlates with DNA methylation of pancreas development genes. Plos One. 2015;10:e0128814. 26039411 10.1371/journal.pone.0128814 PMC4454596

[25] Sidaway P Colorectal cancer: CpG island methylation indicates inferior survival outcomes. Nat Rev Clin Oncol. 2016;13:464–5. 10.1038/nrclinonc.2016.107 27377133

[26] Cha Y, Kim KJ, Han SW, et al Adverse prognostic impact of the CpG island methylator phenotype in metastatic colorectal cancer. Br J Cancer. 2016;115:164–71. 27310704 10.1038/bjc.2016.176 PMC4947699

[27] Willemsen MH, Ba W, Wissink-Lindhout WM, et al Involvement of the kinesin family members KIF4A and KIF5C in intellectual disability and synaptic function. J Med Genet. 2014;51:487–94. 24812067 10.1136/jmedgenet-2013-102182

[28] Kimura K, Wakamatsu A, Suzuki Y, et al Diversification of transcriptional modulation: large-scale identification and characterization of putative alternative promoters of human genes. Genome Res. 2006;16:55–65. 16344560 10.1101/gr.4039406 PMC1356129

[29] Vale RD The molecular motor toolbox for intracellular transport. Cell. 2003;112:467–80. 12600311 10.1016/s0092-8674(03)00111-9

[30] Salinas RE, Ogohara C, Thomas MI, et al A cellular genome-wide association study reveals human variation in microtubule stability and a role in inflammatory cell death. Mol Biol Cell. 2014;25:76–86. 24173717 10.1091/mbc.E13-06-0294 PMC3873895

[31] Ey B, Eyking A, Klepak M, et al Loss of TLR2 worsens spontaneous colitis in MDR1A deficiency through commensally induced pyroptosis. J Immunol. 2013;190:5676–88. 23636052 10.4049/jimmunol.1201592 PMC3659955

[32] Cucchiarelli V, Hiser L, Smith H, et al Beta-tubulin isotype classes II and V expression patterns in nonsmall cell lung carcinomas. Cell Motil Cytoskeleton. 2008;65:675–85. 18613117 10.1002/cm.20297

[33] Leandro-García LJ, Leskelä S, Landa I, et al Tumoral and tissue-specific expression of the major human beta-tubulin isotypes. Cytoskeleton (Hoboken). 2010;67:214–23. 20191564 10.1002/cm.20436

[34] Mariani M, Zannoni GF, Sioletic S, et al Gender influences the class III and V β-tubulin ability to predict poor outcome in colorectal cancer. Clin Cancer Res. 2012;18:2964–75. 22438565 10.1158/1078-0432.CCR-11-2318

[35] Xiao H, Verdier-Pinard P, Fernandez-Fuentes N, et al Insights into the mechanism of microtubule stabilization by taxol. Proc Natl Acad Sci U S A. 2006;103:10166–73. 16801540 10.1073/pnas.0603704103 PMC1502429

