# Supplementary material for: Discovery and Validation of Methylation Biomarkers for Ulcerative Colitis Associated Neoplasia
Source: Inflamm Bowel Dis. 2018 May 14;24(7):1503–9. doi: 10.1093/ibd/izy119 (PMC6176894; doi:10.1093/ibd/izy119)
Supplement: Supplementary Material [file izy119_suppl_supplementary_materials.docx]

**Supplementary results for Beggs et al**

As no immunohistochemistry was possible on tissue sections because of limited tissue from biopsy specimens, an exploratory analysis of the TCGA COAD (colorectal cancer) cohort was carried out using CBioPortal ([www.cbioportal.org](http://www.cbioportal.org) ). Methylation values (determined from the Human Methylation 450 array) were plotted against mRNA expression (as determined by RNA seq). No correlation was found between the two. An examination of available protein level data from the Protein Atlas ([www.proteinatlas.org](http://www.proteinatlas.org)) determined that there was global expression from TUBB6 for two different antibodies (HPA043640, HPA046280) across all tissue types. However, this expression was lost in the majority of solid tumours examined using this antibody.

**Supplementary figure 1: Scatter plot of mRNA expression vs. methylation of TUBB6.**


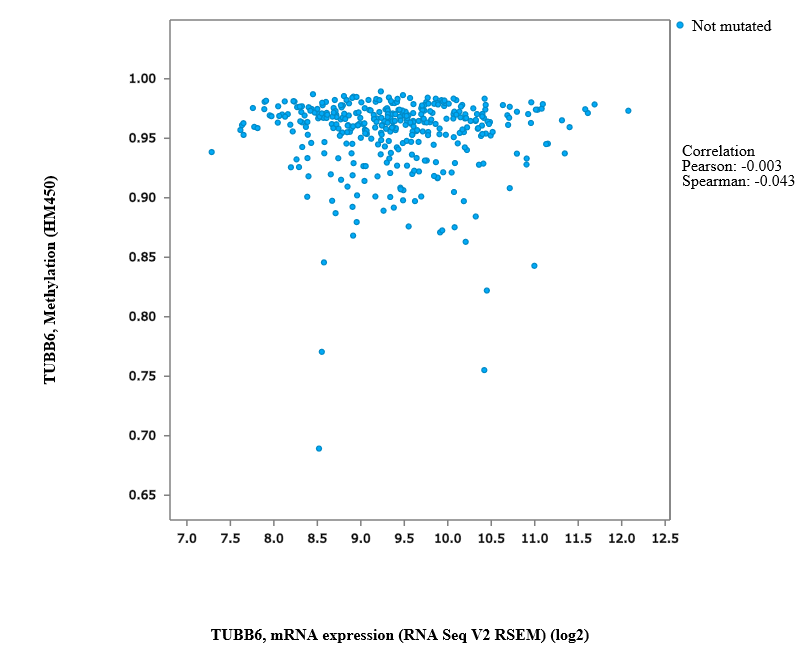


**Supplementary figure 2: ROC curve for TUBB6 for predicting dysplasia at 17% threshold methylation.**
